# Rehabilitation of Children with Hemiparesis: A Pilot Study on the Use of Virtual Reality

**DOI:** 10.1155/2013/695935

**Published:** 2013-10-02

**Authors:** Ivana Olivieri, Matteo Chiappedi, Paolo Meriggi, Marcella Mazzola, Attilia Grandi, Lucia Angelini

**Affiliations:** Don Carlo Gnocchi ONLUS Foundation, Piazzale Morandi 6, 20121 Milan, Italy

## Abstract

*Background*. A wide range of treatments have been used to improve upper arm motor performances in children with congenital hemiplegia. Recent findings are suggesting that virtual reality based intervention could be a promising tool also in pediatric rehabilitation. *Methods*. Six patients with congenital hemiplegia (age: 4–16 years) were recruited among those treated in the Child Neuropsychiatry and Rehabilitation Unit of the IRCCS “Santa Maria Nascente” (Milan, Italy), for a preliminary investigation about using nonimmersive virtual reality for upper limb rehabilitation. Ten sessions using VRRS system (Khymeia, Padova, Italy) were weekly administered as a part of the rehabilitative treatment. Melbourne Assessment of Unilateral Limb Movement, Ashworth Scale, and Arm's PROM were selected as main outcome measures. At the end of treatment, participants filled in an ad hoc satisfaction questionnaire. *Results*. All subjects completed the proposed treatment, and they also gave a positive judgment regarding this rehabilitative method. Melbourne score increased in all patients. *Conclusion*. Our findings seem to support the evidence that VR treatment could be a promising and engaging tool for pediatric rehabilitation. However, the limited size of the population and the small number of sessions require further investigations and RCTs to confirm our positive results.

## 1. Introduction

Cerebral palsy is a relatively common motor disability in childhood, with prevalence of 2.18 per 1000 live births. Congenital hemiplegia accounts for 38% of cases [1]: these patients present a unilateral impairment with a major involvement of the upper limb. Spasticity, weakness, and reduced joint range of motion (ROM), associated with a decrease in speed and accuracy of movements, often lead to a severe reduction in using the paretic arm in everyday activities. A wide range of physical, pharmacological, and surgical treatments have been proposed and used to improve upper arm motor performances [2]. Most physical therapies last for years and/or require a level of intensity of treatments which may reduce children's interest and compliance over time. Clinical work suggests on the other hand that the use of meaningful and engaging activities is a key element for a successful therapy, especially with young subjects [3]. 

Virtual reality (VR) based treatment might play an important role in pediatric rehabilitation. VR is a computer based artificial environment, presented to the user through appropriate sensory stimulations. Subjects interact with the virtual environment through a number of sensing devices (i.e., keyboards, mice, motion sensors, cameras, etc.), receiving a continuous feedback, which is usually visual and auditory, but might also be kinesthetic. 

A recent Cochrane review reported that VR based intervention is more effective than conventional therapy in recovering upper limb functionalities in poststroke adult patients [4]. Although few studies have focused on the use of VR in children, recent findings suggested that it could be an engaging and promising tool for pediatric rehabilitation [5, 6] and in particular for children with unilateral upper limb impairment [7, 8]. 

Aim of the present work is preliminary investigation about feasibility, effectiveness, and engagement in using nonimmersive Virtual reality for upper limb rehabilitation in children with congenital hemiplegia.

## 2. Methods

Participants were recruited among those treated with traditional neuromotor therapy at the Child Neuropsychiatry and Rehabilitation Unit of the Don Carlo Gnocchi Foundation, IRCCS “Santa Maria Nascente” (Milan, Italy). We included subjects with congenital hemiplegia, Intelligence Quotient higher than 70, absence of severe hypoacusia and hypovision, and absence of botulinum toxin and/or surgical treatment in the six past months. 

Six patients were enrolled, four males and two females (mean age 8, 6 years; DS 4,5 years), equally divided according to the hemiplegic side (three left, three right side).

According to Gross Motor Function Classification System, all children are in Level I [9]. The Gross Motor Function Measure Scale (GMFM) [10] was administered before the treatment in order to assess gross motor functions (as detailed in Table 1).

Ten 45-minute sessions were administered to each child, once per week, as part of the ongoing rehabilitative treatment: every child received one session of traditional physiotherapy and one VR based treatment per week without increasing the number of treatments per week. 

The VR system used was a nonimmersive commercially available product, VRRS (Khymeia, Padova, Italy), already used in adult rehabilitation [11]. Subject's movements were detected by means of G4 (G4—Polhemus, Colchester, USA), a compact electromagnetic based wireless motion tracker, that measures the position and orientation in space, relative to initial conditions. The tracker is composed by an electronic unit (sensor hub) connected to up to three small sensors that might be worn by subjects. 

**Figure 1:**
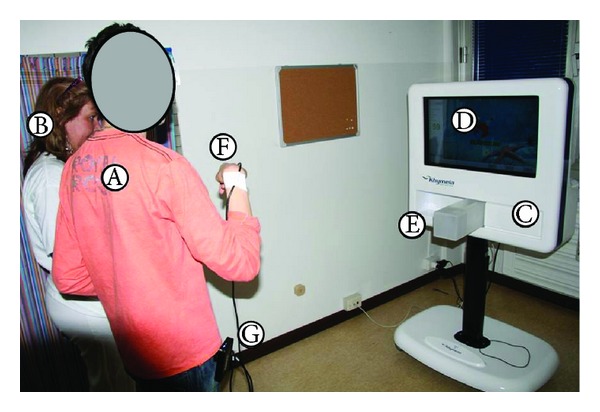
The actual setup during an exercise. (A) Participant, (B) PT, (C) computer, (D) screen, (E) antenna, (F) sensor placed on the hand, and (G) G4 sensor hub.

**Figure 2:**
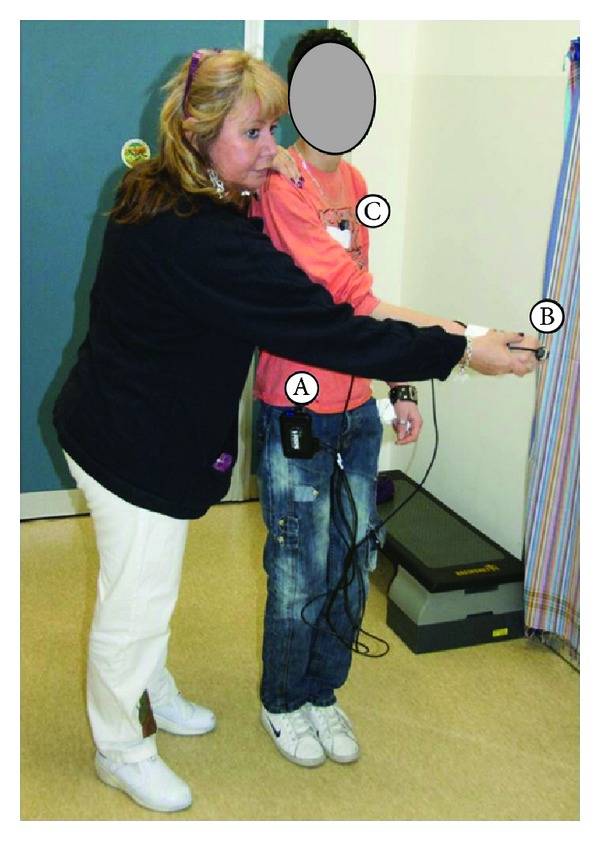
PT showing the user how to interact with the virtual environment. (A) G4 sensor hub, (B) sensor placed on the hand, and (C) sensor placed on the trunk.

**Figure 3:**
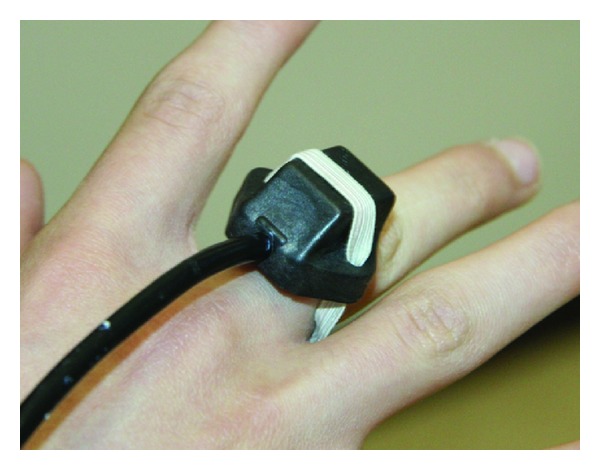
Closeup picture of the sensor placed on the hand.

In the actual setup of the exercises performed by participants (reported in Figures 1 and 2), we used only two sensors, one placed on the back of the hand (Figure 3) and one on the trunk. The sensor on the hand was used to move a simple object (a sphere) within the virtual environment, while the sensor on the trunk was meant to provide a visual feedback to participants, helping them to maintain the upright position, without excessive bending. Cabling from sensors to hub was firmly taped on the forearm and on the subjects' clothes (see Figure 2).

Exercises administered by means of VRRS had the aim of improving reaching and tracking movements of the paretic arm. Since the available 3D-scenarios were originally designed for adult patients, in cooperation with Khymeia developers, we adapted two scenarios and created four new virtual environments more suitable to children for our rehabilitation goals.

EX-1: the subject was asked to grab colored cubes and spheres and to stack them up according to a specific sequence reported on a side of the screen.
EX-2: the subject had to move a colored ball following a reference curved trajectory, painted on the screen.
EX-3: the subject had to move a colored ball following reference straight trajectories in both vertical and horizontal directions, painted on the screen.
EX-4: the subject was asked to hit a frog appearing in random places on the top and around a virtual castle placed in the center of the screen.
EX-5: the subjects had to grab a quickly moving fish in a sort of underwater environment.
EX-6: the exercise was developed to mimic a common everyday activity: to tide up a sort of virtual bedroom by picking up all the toys scattered on the carpet and putting them in a basket.

In order to improve children's attention and motivation, specific acoustic and visual cues were presented during each scenario. These cues helped the subjects to understand if they were performing well and/or if they reached the specific goal of the exercise. Particular attention was paid to the graphics in order to have visually appealing environments and to minimize the crowding effect which could reduce the attention level in the specific activity.

All six scenarios were proposed in each session to every participant. Physiotherapist (PT) was actively involved during the VR sessions, to support children and to adapt the parameters and tailor them according to the characteristics and capabilities of participants.

Each subject was evaluated by the same PT before and after the VR rehabilitation, while another PT administered the VR sessions. 

The pre- and postassessments were composed of the following.

- Melbourne Assessment of Unilateral Upper Limb Function [12].
- Modified Ashworth scale [13].
- Upper limb passive range of motion (PROM).

The Melbourne Assessment is an evaluation tool used to objectively measure unilateral upper limb function in children. The Melbourne Assessment includes 16 test items where the child is required to perform a specific (dominant) upper limb task with verbal instruction and in some instances following demonstration by the PT. The dominant and nondominant hand can be assessed. The items include reach (various planes, palm to bottom and forehead to neck), grasp, drawing, release (crayon and pellet), manipulation, pointing, pronation/supination, hand to hand transfer hand to mouth and down. The equipment is positioned so that the child is provided with visual cues (e.g., a “smiley face” switch is positioned by the PT in front for forwards reach, and to the side for reach sideways to elevated position). The child's test performance is recorded by videotape to enable posttest scoring. Scoring criteria have been individually defined for each test item. A score sheet with 37 subscores is used to record results. Each subscore is rated on a 3-, 4-, or 5-point rating scale.

The Modified Ashworth Scale (MAS) measure spasticity. During the administration, the examiner passively moves the joint being tested and rates the perceived level of resistance in the muscle groups opposing the movement. This scale is single-item measures ranging from 0 to 4, where 0 indicates no increase in muscle tone and 4 indicates that the affected part is rigid in flexion or extension. 

Passive joint ranges of motion (PROM) of the arm were measured using a goniometer.

Finally, at the end of the VR based treatment period, an ad hoc satisfaction questionnaire was administered to each child. This consisted of seven items, allowing participants to describe if they enjoyed the treatment, if they experienced difficulties, fatigue, or pain in performing the activities proposed, and if they would like to repeat the experience.

For the first six items, the child could choose among three possible answers indicated by specific “emoticons” (representing “yes,” “so-so,” and “no”). The last item was an open-ended question in which participants were asked to indicate if they experienced any pain or discomfort and where (a simplified sketch of a human body was also available as a help for the younger ones).

The study received the approval of the local Ethical Committee. Parents gave a written consent to the study. 

### 2.1. Statistical Analysis

We could not assume Melbourne scores to be normally distributed (at baseline: kurtosis: −1.795; asymmetry: −0.682). The Wilcoxon test for paired samples was therefore used to compare scores before and after treatment. The analysis was performed using SPSS version 15 (SPSS Inc., Chicago, USA).

## 3. Results

All participants completed the VR rehabilitation sessions, none of them reported negative side effects. The PT, who supported and actually administered the VR treatments, did not report difficulties in setting up the system and while participants were performing exercises. 

Results of pre- and posttreatment evaluations are detailed in Table 2. 

Melbourne scores increased in all participants after treatment; this change was statistically significant (Wilcoxon test for paired samples: *Z* = 2.201, *P* = 0.028). No statistically significant changes occurred in PROM and Ashworth scales, although some values indicate an increase in the performances at the end of the treatment. 

As to the satisfactory questionnaire, all children stated that they enjoyed the treatment (five children answered “yes” and one “so-so”). Four children reported no difficulties in performing the proposed activities, while the other two required, in the initial sessions, some help from the PT but quickly managed to perform the proposed tasks on their own. Four participants reported that they would like to repeat the experience. Three subjects reported a certain degree of fatigue limited to the initial sessions of the treatment.

## 4. Discussion

Aim of this study was to initially evaluate the feasibility of using a VR system in rehabilitation of upper limb in children with congenital hemiplegia. 

There are many VR systems designed to provide high sense of presence in the virtual environment (semiimmersive and immersive VR—Kalawsky [14]). They usually require head mounted displays (HMDs), suitable 3D goggles, or several screens surrounding the subject. However, their complexity (i.e., using HMDs), high costs, and most of all possible negative side-effects (i.e., cybersickness) [6] make them poorly suitable for a wide use in pediatric rehabilitation. 

On the other hand, an increasing number of VR systems, based on off-the-shelf devices (usually game-consoles), have been proposed. These types of solutions have some benefits: ready availability, low cost, fairly intuitive mode of interaction, and an engaging format [8]. However, these systems present some great disadvantages. Since they are designed for users without motor or cognitive disabilities, they cannot be adapted to meat particular needs, and, most of all, they cannot provide quantitative information about users' performance. Moreover, many children with intellectual disabilities will not readily identify the goal of the activity and will therefore be unable to set up an appropriate motor response [8].

Even with the above described limitations, VR based systems offer a range of flexible treatment solutions for PTs (a single device might be used for many different exercises, depending on the software) and might enhance the motivation in children towards the therapeutic exercise. Thus, there is a growing interest in solutions that might offer the appeal of video-games, the flexibility in adapting to users' abilities, and the possibility to derive quantitative information about the exercises performed.

To meet these three features, we adapted some scenarios of a commercially available VR system (VRRS by Khymeia). Since it is based on electromagnetic principle, the system measures the position and orientation in space, relative to initial conditions, of up to three small sensors. This provides, in a work volume of about eight cubic meters, drift-free highly accurate and repeatable tracking data. This is an important difference with respect to most of the other sensing devices derived from gaming consoles, where these data are calculated and not measured.

A high degree of motivation and involvement in performing proposed activities emerged during VR based sessions; no child refused the treatment, and no one reported negative side effects. Patients gave positive judgments in the satisfaction questionnaire: all of them enjoyed the treatments, and most of them were interested in repeating a similar experience. They expressed no major difficulty in understanding the tasks. A certain degree of fatigue in the initial sessions, reported by a few patients, was probably due to a possible increase in the physical involvement in this novel form of rehabilitation.

There was an overall improvement in the use of the paretic arm, as shown by the increased Melbourne scores. Of course, the statistical significance of the change in Melbourne scores should be considered with caution given the low number of patients in our sample [15]; nevertheless, this result is still interesting compared to traditional rehabilitation considering the small number of performed sessions.

In some patients, there was also a reduction of muscular hypertonus and an increase of shoulder mobility (PROM), but further studies will be required to properly foster these results, especially to clarify if and how a VR based treatment might lead to a lasting increase in motor performances. 

Our study has however some limitations. First of all, we did not gather information about the VR treatment alone, since participants did not interrupt their usual physiotherapeutic treatments. This is a major limitation and should be further clarified by a controlled study, which will however be made possible by the preliminary findings of this pilot study.

Moreover, in this study we did not evaluate if the increase in motor performances might represent an overall and lasting increase in the subjects' ability to perform daily activities and not simply a change of motor skills limited to the testing conditions.

In our study, there was not a specific investigation about the importance and the role of the PTs, although the collected comments clearly underline that their presence was of key importance for the best engagement and for its supportive and mediatory functions.

Future randomized controlled studies on larger samples are needed to overcome these limitations and adequately investigate efficacy of VR based treatment in hemiplegic upper limbs rehabilitation in children. 

## Figures and Tables

**Table 1:** Description of the study group.

Subjects	1	2	3	4	5	6
Sex	M	M	M	M	F	F
Age	11	5	6	16	5	7
Side of motor impairment	Dx	Dx	Sx	Sx	Sx	Dx
GMFM (Total)	95	83	96	93	100	93
(A) lying and rolling	100	98	100	94	100	100
(B) crawling and kneeling	93	71	100	93	100	100
(C) sitting	97	93	100	92	100	100
(D) standing	95	79	95	97	100	90
(E) walking, running, jumping	90	76	86	88	100	78

**Table 2:** T0 and T1 assessment results.

Subjects	1		2		3		4		5		6	
	T0	T1	T0	T1	T0	T1	T0	T1	T0	T1	T0	T1
PROM (°)												
Shoulder												
Flexion	160	170	170	180	160	175	155	155	170	170	170	180
Abduction	140	150	120	130	120	140	100	120	115	115	140	150
Elbow												
Flexion	145	145	150	150	140	140	140	140	145	145	145	145
Estension	165	170	−5	−5	175	180	165	175	−5	−5	170	180
Wrist												
Flexion	90	90	90	90	90	100	90	90	90	90	90	90
Estension	20	20	75	90	80	90	60	75	80	90	80	80
MAS												
Shoulder	3	1	1	1	1	1	1	0	1	1	1	0
Elbow	4	2	1	1	2	1	1	1	0	0	1	0
Wrist	4	3	1	1	1	1	2	1	0	0	0	0
Melbourne* (%)	40	49	36	52	80	87	82	93	94	97	99	100

*Wilcoxon test for paired samples: *Z* = 2.201, *P* = 0.028.
